# Nanostructured SL9-CpG Lipovaccines Elicit Immune Response for the Treatment of Melanoma

**DOI:** 10.3390/ijms20092207

**Published:** 2019-05-05

**Authors:** Li-Min Mu, Lei Liu, Rui Liu, Ya-Fei Du, Qian Luo, Jia-Rui Xu, Ying Xie, Wan-Liang Lu

**Affiliations:** State Key Laboratory of Natural and Biomimetic Drugs, Beijing Key Laboratory of Molecular Pharmaceutics and New Drug System, and School of Pharmaceutical Sciences, Peking University, Beijing 100191, China; liminmu@bjmu.edu.cn (L.-M.M.); liulei@bjmu.edu.cn (L.L.); liuruibjmu@163.com (R.L.); duyafei77@163.com (Y.-F.D.); Luoqian7777@126.com (Q.L.); xujiarui228@foxmail.com (J.-R.X.); bmuxieying@bjmu.edu.cn (Y.X.)

**Keywords:** lipovaccines, cytotoxic T cells, melanoma, antigen peptide, cancer immunotherapy

## Abstract

Antigen peptides and adjuvants have been extensively investigated for cancer immunotherapy, and they are expected to elicit specific immune responses for cancer treatment. However, the anti-cancer efficacy of antigen peptide and adjuvant-based cancer vaccines has been limited due to the inefficient delivery to draining lymph nodes after administration. Therefore, it is necessary to develop a suitable delivery system to transport antigen peptides and adjuvants. Here, we report a novel type of nanostructured lipovaccines for the treatment of melanoma by delivering antigen peptide (SL9) and oligodeoxynucleotide adjuvant (CpG) to the lymphatic vessels and to the draining lymph node. The SL9-CpG lipovaccines were characterized using dynamic laser scattering (DLS) and transmission electron microscopy (TEM). The lymph uptake, immune response elicitation and treatment effects were evaluated on melanoma-bearing C57BL/6 mice using flow cytometry (FCM), enzyme-linked immunosorbent assay (ELISA) and tumor inhibitory efficacy. The SL9-CpG lipovaccines were uniform with a nanoscale size (~70 nm), had high encapsulation efficiency, and exhibited effective lymph uptake, resulting in activation of specific cytotoxic CD8+ T cells, and release of IFN-γ, and a robust inhibition of tumor growth. Therefore, the nanostructured SL9-CpG lipovaccines offer a promising strategy for melanoma treatment.

## 1. Introduction

Cancer vaccines mainly focus on stimulating and enhancing the immune system to recognise cancer cells, therefore killing cancer cells [1]. Over the past decades, a variety of antigen peptides have been explored and extensively developed to engender cytotoxic T cell-mediated tumor attacks [2]. However, the anti-cancer efficacy of antigen peptide-based cancer vaccines has been limited in animal and clinical trials due to the inefficient delivery of antigen peptides and adjuvants to draining lymph nodes [3,4]. A series of studies have proven that nanoparticles (including liposomes, micelles, nanotubes, etc.) with particle sizes in the range of 10 nm to 100 nm could be quickly and efficiently taken up by lymphatic vessels [5,6]. Consequently, the nanoparticles could transport antigens to the draining lymph node through the vasculature and deliver antigens to dendritic cells, which present the antigen to lymphocytes and stimulate an immune response [7]. Therefore, in this study, we propose a type of nanostructured liposome vaccines, which potentially transport the antigen peptide (SL9) and adjuvant (cholesterol-CpG) to lymph nodes to stimulate an immune response, thus eliminating melanoma cells.

Liposomes are nanoscale vesicles with an aqueous core and bilayer membranes enclosed by phospholipids or sphingolipids, and are capable of incorporating hydrophobic, amphipathic and hydrophilic agents [8]. They are widely used for drug delivery due to their biocompatibility and biodegradability, with increased efficacy and reduced systemic toxicity [9]. Liposomes are capable of delivering antigens to lymph nodes and of protecting the encapsulated antigens from enzymatic degradation.

SVYDFFVWL (SL9) is a peptide derived from tyrosinase-related protein 2 (TRP2) residues 180–188 and has been identified as the primary epitope of TRP2, recognized by anti-B16 melanoma cytotoxic T lymphocytes (CTLs) [10]. SL9 is presented by the major histocompatibility complex class I molecules on matured dendritic cells (DCs) to T lymphocytes for stimulating antigen-specific CTLs, i.e., antigen-specific CD8+ T cells, which mediate antigen-specific lysis of cancer cells by releasing cytolytic granule proteins, such as granzymes and perforin [11]. SL9 peptide has been widely studied with respect to stimulating an immune response for cancer therapy. However, SL9 is difficult to formulate in an aqueous solution, and the inefficient delivery of SL9 to lymph nodes, especially to antigen presenting cells, hinders its efficacy [12]. Therefore, the development of an effective delivery system for SL9 is essential. In addition, CpG oligodeoxynucleotide (ODN) is a short single-stranded synthetic DNA molecule, in which C refers to cytosine triphosphate deoxynucleotide, G refers to a guanine triphosphate deoxynucleotide, and *p* refers to the phosphodiester link between consecutive nucleotides [13]. CpG is able to stimulate Toll-Like Receptor 9 (TLR9), thus, activating dendritic cells to initiate a cascade of innate and adaptive responses [14]. Therefore, it can be provided as an adjuvant to enhance the anti-cancer efficacy of a variety of cancer treatments [15].

The objectives of this study are to develop a novel type of lipovaccines by co-delivery of antigen peptide SL9 and cholesterol-CpG oligonucleotide, and to evaluate the delivery efficiency of SL9 to lymph nodes, the activation effect of antigen-specific CD8+ T cells, and the treatment efficacy for melanoma in mice. The present study may provide a new formulation strategy and experimental evidence for cancer immunotherapy.

## 2. Results

### 2.1. Characterization of SL9-CpG Lipovaccines

**Figure 1:**
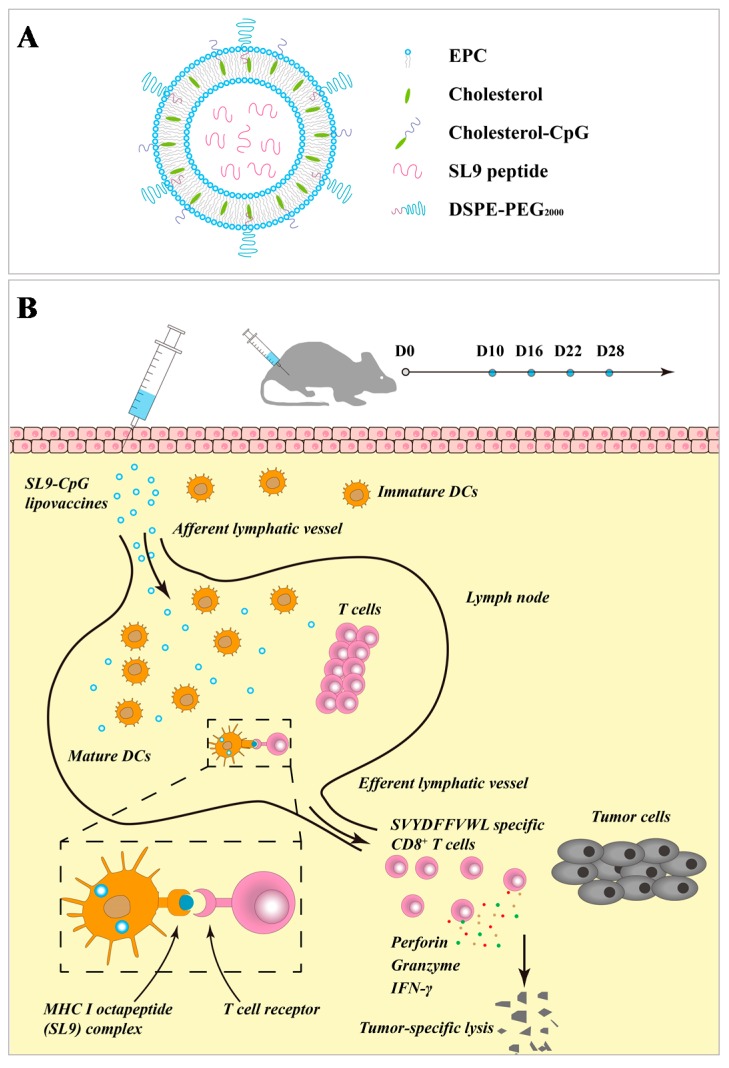
Illustrations of structure and action mechanism of SL9-CpG lipovaccines. Schematic presentation for the structure of SL9-CpG lipovaccines (**A**). Illustration for action mechanism of SL9-CpG lipovaccines (**B**). EPC, egg phosphatidylcholine; CpG, cytosine triphosphate deoxynucleotide-phosphodiester-guanine triphosphate deoxynucleotide; DCs, dendritic cells; MHC I, major histocompatibility complex I.

To fabricate the SL9-CpG lipovaccine, SL9 was encapsulated into the inner core of the liposomes (Figure 1A). Cholesterol-CpG was inserted into the lipophilic bilayer and the CpG component was left on the liposome exterior. The SL9-CpG lipovaccine was designed for subcutaneous injection to be drained into afferent lymphatic vessels to take effect. Briefly, the action is illustrated as the process whereby the lipovaccines are internalized by dendritic cells, which further present the antigen to T cells via MHC class I pathway (Figure 1B).

**Figure 2:**
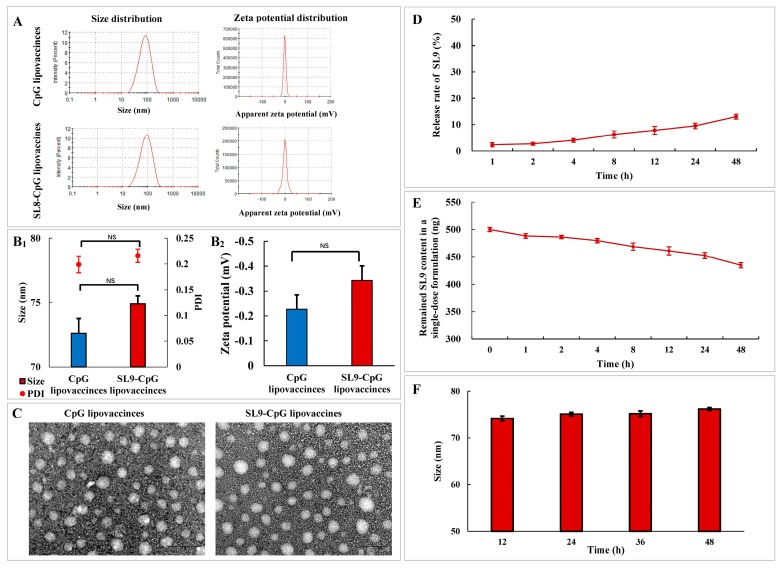
Characterization of SL9-CpG lipovaccines. Size and zeta potential distributions of CpG lipovaccines and SL9-CpG lipovaccines by dynamic laser scattering (DLS) (**A**). Size and PDI (**B1**) and zeta potential (**B2**) statistical results of CpG lipovaccines and SL9-CpG lipovaccines. PDI, polydispersity index. Data are presented as mean ± standard deviation (*n* = 3). NS, not significant. Transmission electron microscope (TEM) images of CpG lipovaccines and SL9-CpG lipovaccines (**C**). Scale bar, 100 nm. Release rate of SL9-CpG lipovaccines in serum containing culture medium (**D**). Loading stability of a single-dose formulation of SL9-CpG lipovaccines in the phosphate buffered saline containing 10% fetal bovine serum (pH 7.4, 37 °C) (**E**). Particle stability of a single-dose formulation of SL9-CpG lipovaccines in phosphate buffered saline (pH 7.4, 37 °C) **(F**). Data are presented as mean ± standard deviation (*n* = 3).

The size, polydispersity index (PDI) and zeta potential were characterized. The average size of SL9-CpG lipovaccines was approximately 74.91 ± 0.62 nm with a narrow polydispersity index (<0.25). The zeta potential was approximately −0.343 ± 0.022 mV (Figure 2A, 2B1 and 2B2). The results from transmission electron microscope (TEM) imaging indicated that both the CpG lipovaccines and SL9-CpG lipovaccines (Figure 2C) were uniform and round with smooth surfaces. The encapsulation efficiency of SL9 in the SL9-CpG lipovaccines was determined using BCA assay. The correlation between the concentration of SL9 peptide and absorbance was A542 nm = 0.0766C + 0.0219 (r = 0.9993). The encapsulation efficiency of SL9 in the SL9-CpG lipovaccines was 82.17 ± 1.36%, and the loading efficiency of SL9 in the SL9-CpG lipovaccines was 4.08 ± 0.07%. The release rates of SL9 from the SL9-CpG lipovaccines in the initial 48 h were all below 20% in the phosphate buffered saline containing blood components (pH 7.4, 37 °C) (Figure 2D). The remaining loading contents of SL9 in a single-dose formulation of SL9-CpG lipovaccines decreased from 500.15 ± 3.81 ng to 452.45 ± 5.25 ng in the initial 24 h, and reduced to 434.85 ± 4.76 ng in the initial 48 h in the phosphate buffered saline containing blood components (pH 7.4, 37 °C) (Figure 2E). The results indicated that above 90% of the SL9 remained in the initial 24 h, and above 87% of the SL9 remained in the initial 48 h, in the single-dose formulation of SL9-CpG lipovaccines. The particle sizes of a single-dose formulation of the SL9-CpG lipovaccines were 75.20 ± 0.60 nm at 24 h, and 76.20 ± 0.34 nm at 48 h, in PBS (pH 7.4, 37 °C) (Figure 2F). The results indicated that the SL9-CpG lipovaccines did not aggregate or depolymerize in the initial 48 h (pH 7.4, 7 °C).

### 2.2. Cellular Uptake by Lymph Node Cells

**Figure 3:**
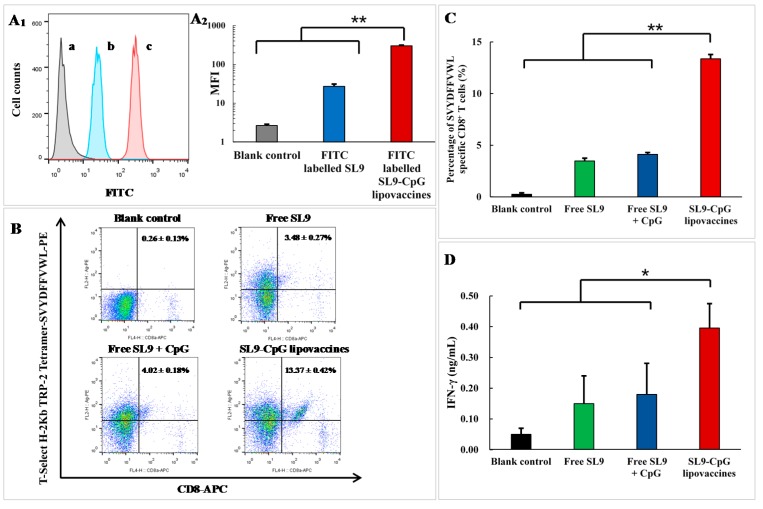
Cellular uptake and immune response in melanoma-bearing mice. Flow cytometry histogram (**A1**) and statistical results (**A2**) of cellular uptake by inguinal lymph node cells: a, blank control; b, fluorescein isothiocyanate (FITC)-labeled SL9 (5 μg/mL SL9-FITC); c, FITC-labeled SL9-CpG lipovaccines (5 μg/mL SL9-FITC, 30 μg/mL CpG). Data are presented as mean ± standard deviation (*n* = 3). ** *p* < 0.01. Scatter diagrams (**B**) and statistical results (**C**) of SVYDFFVWL-specific CD8+ T cells activated by SL9-CpG lipovaccines (5 μg/mL SL9, 30 μg/mL CpG) or controls. Data are presented as mean ± standard deviation (*n* = 3). ** *p* < 0.01. IFN-γ expression levels in peripheral blood by ELISA after immunization with SL9-CpG lipovaccines (5 μg/mL SL9, 30 μg/mL CpG) or controls (**D**). Data are presented as mean ± standard deviation (*n* = 3). *p < 0.05.

The cellular uptake by inguinal lymph node cells was assayed by flow cytometry. After immunization for 24 h by fluorescent probe fluorescein isothiocyanate (FITC)-labeled SL9-CpG lipovaccines (5 μg/mL SL9-FITC, 30 μg/mL CpG) or free FITC-labeled SL9 (5 μg/mL SL9-FITC), the inguinal lymph nodes were obtained and made into single cell suspensions. Fluorescence intensities of the cells were used to indicate the cellular uptake by lymph node cells (Figure 3A and 3A2). The results showed that after subcutaneous injection into the tail base, free SL9 and SL9-CpG lipovaccines were taken up by inguinal lymph node cells, but the uptake efficiency of SL9-CpG lipovaccines was significantly improved compared to that of free SL9-FITC (*p* < 0.01).

### 2.3. Cytotoxic T Lymphocytes Activation Effect

The mice were immunized with SL9-CpG lipovaccines (5 μg/mL SL9, 30 μg/mL CpG) or the controls subcutaneously at the tail four times. On day 6 after the final immunization, the cytotoxic T lymphocyte activation effect by the SL9-CpG lipovaccines or the controls was assayed by detecting the percentage of SVYDFFVWL-specific CD8+ T cells in peripheral blood using flow cytometry. Physiological saline was injected as a blank control. After administration with various formulations, the percentages of SVYDFFVWL-specific CD8+ T cells in peripheral blood were 0.26 ± 0.13% for physiological saline, 3.48 ± 0.27% for free SL9, 4.02 ± 0.18% for the mixture of free SL9 and CpG, and 13.37 ± 0.42% for SL9-CpG lipovaccines (Figure 3B,C).

### 2.4. IFN-γ Release Effect

The expression levels of IFN-γ released from T cells induced by the SL9-CpG lipovaccines or the controls were assayed using enzyme-linked immunosorbent assay (ELISA). After immunization with the SL9-CpG lipovaccines (5 μg/mL SL9, 30 μg/mL CpG) or the controls four times, peripheral blood was collected from melanoma-bearing mice on day 6 after the final immunization. Physiological saline was injected as a blank control. In the blank control group, the expression level of IFN-γ was 0.05 ± 0.02 ng/mL (Figure 3D). The administration with free SL9 alone, or with the mixture of free SL9 and CpG, did not result in significantly elevated expression levels of IFN-γ. Free SL9 resulted in 0.15 ± 0.09 ng/mL and the mixture of free SL9 and CpG resulted in 0.18 ± 0.10 ng/mL of IFN-γ expression in peripheral blood. In contrast, the equivalent SL9-CpG lipovaccines resulted in 0.40 ± 0.08 ng/mL IFN-γ expression in peripheral blood.

### 2.5. Anticancer Efficacy in Melanoma-Bearing Mice

**Figure 4:**
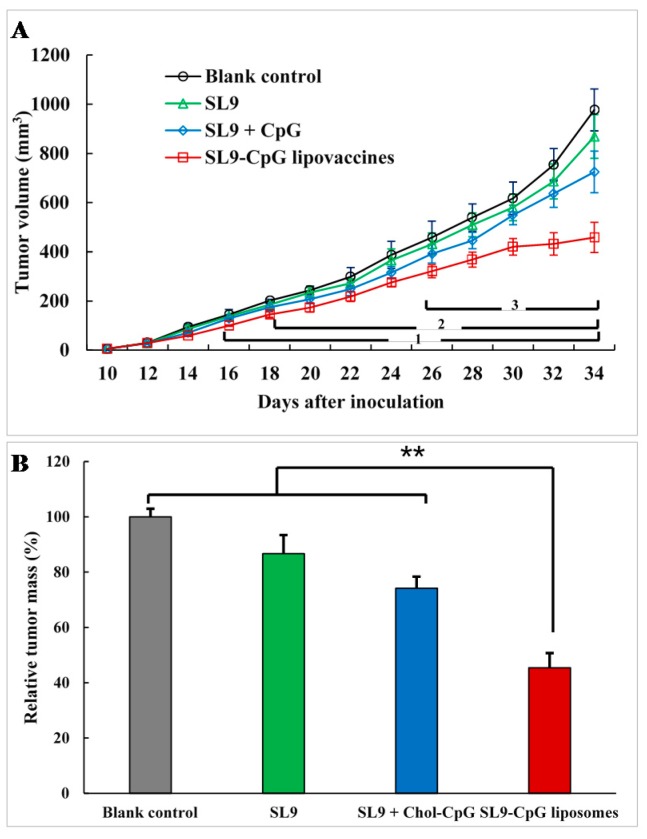
Anticancer effect in melanoma-bearing mice after immunization with SL9-CpG lipovaccines or controls. Tumor volumes after treatment with SL9-CpG lipovaccines (5 μg/mL SL9, 30 μg/mL CpG) or controls (**A**). Data are presented as mean ± standard deviation (*n* = 8). *p* < 0.05; 1, vs. physiological saline; 2, vs. free SL9; 3, vs. free SL9 + CpG. Relative tumor weight (%) after treatment with SL9-CpG lipovaccines (5 μg/mL SL9, 30 μg/mL CpG) or controls (**B**). Data are presented as mean ± standard deviation (*n* = 8). ** *p* < 0.01.

The activation of SVYDFFVWL-specific CD8+ T cells by SL9-CpG lipovaccines could lead to the release of cytokines (IFN-γ), perforin, and granzyme, resulting in lysis of cancer cells. Tumor growth could therefore be inhibited. Xenograft melanoma tumors were measured every two days from day 10 after inoculation and treatment with SL9-CpG lipovaccines (5 μg/mL SL9, 30 μg/mL CpG) or the controls were applied for four times on day 10, 16, 22, and 28. Physiological saline was injected as a blank control. The endpoint of the experiment was set on day 34. Tumor volume curves over time are shown in Figure 4A. The results indicated that free SL9 alone or the mixture of free SL9 and CpG did not show significant inhibition of tumor growth compared to the blank control group, while SL9-CpG lipovaccines resulted in significant tumor growth inhibition (*p* < 0.05) compared to a blank control from day 16, to SL9 group from day 18, and to free SL9 and CpG group from day 24. On day 34 (endpoint), the overall tumor growth inhibition ratios of free SL9, the mixture of free SL9 and CpG, and the SL9-CpG lipovaccines were 15.30 ± 8.67%, 19.06 ± 9.46%, and 64.18 ± 4.78%, respectively. Accordingly, the relative tumor weights on day 34 were 86.64 ± 6.79%, 74.17 ± 4.20%, and 45.43 ± 5.26%, respectively (Figure 4B). The results demonstrated that SL9-CpG lipovaccines had the strongest efficacy in inhibiting tumor growth in melanoma-bearing mice. 

## 3. Discussion

Albeit that comprehensive cancer treatment strategies have achieved evident progress in improving the life quality and the survival prognosis for cancer patients, the overall survival rate of patients after treatments with surgery, chemotherapy and radiotherapy is still limited due to the refractory nature of cancer. Cancer immunotherapy represents a promising strategy for cancer treatment because it kills cancer cells by improving the immune system. However, cancer immunotherapy by cancer vaccines remains a challenging mission. Herein, we reported a novel type of SL9-CpG lipovaccines for the treatment of melanoma by delivering antigen peptide (SL9) and oligodeoxynucleotide adjuvant (CpG) to the lymphatic vessels and to the draining lymph node.

The antigen peptide SL9 (SVYDFFVWL) could activate cytotoxic CD8+ T cells, which are capable of releasing a series of cytolytic granule proteins and killing cancer cells. Therefore, SL9 was selected as the specific antigen to be encapsulated into liposomes to construct the lipovaccines. CpG oligodeoxynucleotide (ODN) could enhance the anti-cancer efficacy of SL9 as an immune adjuvant due to its potent immunostimulatory effects. By conjugating CpG with cholesterol, cholesterol-CpG could be inserted into liposomes. Liposome vaccines, named SL9-CpG lipovaccines, were fabricated by encapsulating SL9 into the inner core and inserting cholesterol-CpG into the lipophilic bilayer with the CpG component left on the exterior (Figure 1A). The SL9-CpG lipovaccines were characterized based on five aspects: morphology, size distribution, zeta potential, encapsulation efficiency, and release rate. The constructed SL9-CpG lipovaccines were uniform with a nanoscale size (~70 nm), nearly neutral zeta potential, and high encapsulation efficiency of SL9 (~80%). Nanoparticles move to the draining lymph nodes in a size-dependent manner after subcutaneous injection, and nanoparticles (e.g., liposomes, nanodiscs, virosomes, etc.) in the size range of 10-100 nm can more often rapidly drain to lymph nodes and are subsequently taken up by resident dendritic cells [5,6]. Therefore, the constructed SL9-CpG lipovaccines met the size requirement for efficient delivery to lymph nodes. The stability of SL9-CpG lipovaccines may potentially affect the immunotherapy efficacy for the treatment of melanoma, especially in the initial 24 h or 48 h after administration. This is because the formulation begins to distribute in lymph nodes or other organs during the initial 24 h to 48 h. The elevated temperature (37 °C) and blood components may lead to the leakage, aggregation or depolymerization of the lipovaccines. Accordingly, the loading stability and particle stability of SL9-CpG lipovaccines during the initial time period were evaluated in the present study. The results suggested that the stable loading efficiency (> 87% in the initial 48 h) of SL9-CpG lipovaccines could ensure the effective dose for treatment after administration in vivo, and the stable particle size of SL9-CpG lipovaccines could guarantee the efficient delivery of the vaccines to the action site. 

Since the lipovaccines were successfully constructed, their delivery efficiency, immunostimulatory effects and anticancer efficacy were investigated on melanoma-bearing mice. After subcutaneous injection into the tail base, FITC-labeled SL9 CpG lipovaccines significantly improved the cellular uptake of SL9 by inguinal lymph node cells compared to free SL9, which mainly resulted from the size distribution of SL9-CpG lipovaccines allowing draining into the lymph nodes [16]. Furthermore, by adding DSPE-PEG2000 into the SL9-CpG lipovaccine bilayer, the retention of the lipovaccines was evidently increased in both primary and secondary lymph nodes, thereby enhancing the immunotherapy effect [17].

In the immunotherapy process, the activation of cytotoxic T lymphocytes plays a key role. By effectively delivering SL9 and CpG to lymph nodes, SL9-CpG lipovaccines were internalized by dendritic cells in the lymph nodes, and dendritic cells presented the antigen to T cells via the MHC class I pathway [18] (as illustrated in Figure 1B). The T cell receptors (TCR) on T cells recognized the antigen presented by the dendritic cells, and T cells were therefore stimulated into SVYDFFVWL-specific cytotoxic CD8+ T cells. The activated cytotoxic CD8+ T cells could release cytolytic granule proteins, such as granzymes and perforin, which induced apoptosis by cleaving critical substrates and activating the caspase family of cysteine proteases, leading to lysis of cancer cells [19,20]. IFN-γ were also released by cytotoxic CD8+ T cells and inhibited cancer cells by inducing apoptosis and suppressing proliferation, and by increasing the sensitivity of cancer cells to the cytolytic activity of cytotoxic CD8+ T cells via the upregulation of MHC class I molecules [21]. Meanwhile, CpG promoted the immune response via interaction with Toll-Like Receptor 9 (TLR9) on dendritic cells, thus enhancing the activation of cytotoxic T cells [22,23]. Consequently, the growth of B16F10 cell-derived melanoma in C57BL/6 mice was significantly inhibited compared to the controls. In addition, the control groups, including free SL9 alone and the mixture of free SL9 and CpG, did not exhibit significant efficacy in activating T cells, release of IFN-γ or inhibition of tumor growth, which was attributed to the inability of free SL9 and free CpG to drain to lymph nodes.

In conclusion, we developed a novel type of nanostructured SL9-CpG lipovaccines, which were able to effectively deliver antigen peptide SL9 and CpG oligonucleotide to lymph nodes, to activate SVYDFFVWL-specific cytotoxic CD8+ T cells, and to induce the release of IFN-γ. The SL9-CpG lipovaccines exhibited a robust inhibition to tumor growth in melanoma-bearing mice. Therefore, the nanostructured SL9-CpG lipovaccines offer a promising strategy for melanoma treatment.

## 4. Materials and Methods

### 4.1. Materials

DSPE-PEG2000 was purchased from NOF Corporation (Kanagawa, Japan), SL9 peptide (SVYDFFVWL) and SL9-FITC (SVYDFFVWL-FITC) were synthesized by Hefei Bankpeptide Company Ltd. (Hefei, China), and cholesterol-CpG ODN was purchased from Sangon Company (Shanghai, China). BCA kit was purchased from Beyotime (Beijing, China). The ouse PBMC isolation kit was purchased from Solarbio (Beijing, China).

Murine melanoma B16F10 cells were obtained from the Institute of Basic Medical Science, Chinese Academy of Medical Science (Beijing, China) and the cells were cultured in Dulbecco’s modified eagle medium (DMEM) supplemented with 10% fetal bovine serum (FBS), 100 U/mL penicillin, and 100 μg/mL streptomycin under an atmosphere of 5% CO2 at 37 °C. Purified anti-mouse CD16/32 antibody, APC anti-mouse CD8α antibody, and the mouse IFN-γ ELISA kit were purchased from Biolegend (Beijing, China). T-Select H-2Kb TRP-2 Tetramer-SVYDFFVWL-PE was purchased from MBL (Beijing agent, China).

### 4.2. Construction of SL9-CpG Lipovaccines

Two types of lipovaccines were prepared, including CpG lipovaccines, and SL9-CpG lipovaccines. Briefly, to prepare SL9-CpG lipovaccines, egg phosphatidylcholine (EPC), cholesterol, cholesterol-CpG, and DSPE-PEG2000 were dissolved in dichloromethane at a molar ratio of 70:20:5:5 in a pear-shaped bottle. The solvent was then removed using a rotary vacuum evaporator, and the lipid film was hydrated with HEPES buffer solution (HBS, 25 mM HEPES, 150 mM NaCl) containing SL9 (lipids: SL9 = 20:1, molar ratio) by sonication in a water bath for 5 min. Afterwards, the suspensions were treated with an ultrasonic cell disruptor for 10 min (200 w), transferred into regenerated cellulose dialysis tubing (MWCO, 8000–14,000 Da), and dialyzed in HBS to exclude free SL9 for 24 h. CpG lipovaccines were prepared following the above procedures without adding SL9 in the hydration procedure. In addition, FITC-labeled SL9 CpG lipovaccines were prepared following the above procedures with SL9-FITC added in place of SL9.

### 4.3. Characterization of SL9-CpG Lipovaccines

The lipovaccines were observed using a transmission electron microscope (JEM-1400, JEOL Ltd., Tokyo, Japan). The size and zeta potential of the lipovaccines were measured using dynamic laser scattering (DLS) with a Nano Series Zenith 4003 Zetasizer (Malvern Instruments Ltd., Malvern, UK). 

The encapsulated efficiency of SL9 was measured using BCA assay according to the manual. Briefly, a series of SL9 standard solutions (0, 0.025, 0.05, 0.1, 0.2, 0.3, 0.4, and 0.5mg/mL) were prepared in a 96-well plate. 200 μL BCA working solution was added and incubated at 37 °C for 30 min. The absorbance of each well was measured using a microplate reader (Infinite F50, Tecan Group Ltd., Beijing, China) at a wavelength of 540 nm, and the correlation between the concentration of SVYDFFVWL peptide and absorbance was obtained. The content of SL9 in the lipovaccines and overall SL9 added were obtained by calculation using the correlation above. Encapsulation efficiency = (Content of SL9 in the lipovaccines / Content of overall SL9 added) × 100%. Loading efficiency = (Weight of SL9 in the lipovaccines / Weight of lipovaccines) × 100%.

The in vitro release rates of SL9 from the constructed lipovaccines were performed by dialysis against phosphate buffered saline (PBS, 137 mM NaCl, 2.7 mM KCl, 8 mM Na2HPO4 and 2 mM KH2PO4, pH 7.4) containing 10% fetal calf serum (FBS) at 37 °C. The released SL9 was measured at 1 h, 2 h, 4 h, 8 h, 12 h, 24 h, and 48 h using the BCA assay mentioned above.

The loading stability of SL9-CpG lipovaccines was estimated by measuring the remained SL9 content in a single-dose formulation over time in the phosphate buffered saline containing 10% fetal bovine serum (pH 7.4, 37 °C). Briefly, single doses of SL9-CpG lipovaccines (100 μL, containing 500 ng SL9) were dialyzed against PBS containing 10% FBS (pH 7.4, 37 °C). The remaining SL9 contents in each dialysis bag were measured at 0 h, 1 h, 2 h, 4 h, 8 h, 12 h, 24 h, and 48 h using the BCA assay mentioned above. Each assay was repeated for triplicate. 

The particle stability of SL9-CpG lipovaccines was evaluated by measuring the average size of a single-dose formulation over time in PBS (pH 7.4, 37 °C). Briefly, single doses of SL9-CpG lipovaccines (100 μL, containing 500 ng SL9) were dialyzed against PBS (pH 7.4). The particle sizes of SL9-CpG lipovaccines over time were measured using DLS at 12 h, 24 h, 36 h, and 48 h. Each assay was repeated in triplicate.

### 4.4. Melanoma-Bearing Mouse Model

All animal experiments adhered to the Principles of Care and Use of Laboratory Animals of Peking University and were approved by the Sub-Committee on the Ethics of Experimental Animal Welfare at Medical Ethics Committee of Peking University (approval no. LA2016151, approval date: 24 February 2016). 

The melanoma-bearing mouse model was established by inoculating B16F10 melanoma B16F10 cells (5 × 10^5^ cells each mouse) into the right flank of female C57BL/6 mice (6–7 weeks, Department of Laboratory Animal Science, Peking University, Beijing, China). At day 10 after inoculation, the mice were randomly divided into several groups according to the different experimental conditions.

### 4.5. Cellular Uptake by Lymph Node Cells

To investigate cellular uptake of SL9-FITC, normal female C57BL/6 mice were divided into two groups (three mice in each group) and immunized by subcutaneous injection into the tail base of FITC-labeled SL9-CpG lipovaccines (5 μg/mL SL9-FITC, 30 μg/mL CpG) or the same dose of controls. The doses of SL9-FITC and CpG per mouse were 500 ng and 3 μg, respectively. After 24 h, the mice were sacrificed, and the inguinal lymph nodes were obtained. The lymph nodes were cut into small pieces and added into tubes containing 1 mL DMEM supplemented with 1 mg/mL collagenase IV. After incubation at 37 °C for 30 min, the cell suspensions were passed through a 70 µm nylon mesh and centrifuged for 50 min at 1500× *g* at 4 °C [24]. The cells were suspended in DMEM and the fluorescence intensity was measured by a flow cytometer (FCM; FACScan, Becton Dickinson, San Jose, CA, USA). The excitation and emission wavelengths were set at 488 nm and 530 nm, respectively.

### 4.6. Cytotoxic T Lymphocytes Activation Effect

To evaluate the T cell activation effect, melanoma-bearing mice were divided into four groups (three mice in each group) and immunized by subcutaneous injection into the tail base of SL9-CpG lipovaccines (5 μg/mL SL9-FITC, 30 μg/mL CpG)or equivalent dose of controls, including physiological saline, free SL9, SL9 and cholesterol-CpG on day 10, 16, 22, and 28 after inoculation. The doses of SL9 and CpG per mouse were 500 ng and 3 μg, respectively.

On day 34 after inoculation, peripheral blood was drawn from the tail vein. Peripheral blood mononuclear cells were obtained using the mouse PBMC isolation kit according to the manual. Briefly, 1 mL peripheral blood and 1 mL isolation solution were added into the tube, and centrifuged (1000× *g*) at 4 °C for 30 min. The PBMCs were carefully transported using the pipettor into another tube and washed twice using phosphate buffered saline (PBS pH7.4; 137 mM NaCl, 2.7 mM KCl, 8 mM Na2HPO4 and 2 mM KH2PO4). The PBMCs were incubated with 10 μL purified anti-mouse CD16/32 antibody on ice for 20 min to block the Fc domain of immunoglobulins and then washed with PBS (pH7.4) three times. Afterwards, the PBMCs were sequentially incubated with 5 μL T-Select H-2Kb TRP-2 Tetramer-SVYDFFVWL-PE and 5 μL APC anti-mouse CD8α antibody at 4 °C for 30 min and then washed with PBS (pH 7.4) three times. The fluorescence intensity was measured by a flow cytometer. The excitation and emission wavelengths for PE were set at 488 nm and 533 nm, respectively. The excitation and emission wavelengths for PE and APC were set at 633 nm and 660 nm, respectively.

### 4.7. IFN-γ Release Effect

For enzyme-linked immunosorbent assay (ELISA), peripheral blood was collected by following the procedure above on day 6 after the final immunization and serum was obtained by centrifugation. The levels of IFN-γ in the serum were measured according to the ELISA kit manual. Briefly, standard stock solution or the samples were anti-mouse IFN-γ pre-coated 96-well strip microplate and incubated at room temperature for 2 h while shaking at 200 rpm. Mouse IFN-γ detection antibody solution was added to each well and the samples were incubated at room temperature for 1 h while shaking. Avidin-HRP A solution was added to each well and the samples were incubated at room temperature for 30 min while shaking. Substrate solution and stop solution were added to each well sequentially. The absorbance of each well was measured using a microplate reader within 30 min at a wavelength of 450 nm.

### 4.8. Anticancer Efficacy in Melanoma-Bearing Mice

To study anticancer efficacy, melanoma-bearing mice were divided into four groups (eight mice in each group) and immunized by subcutaneous injection into the tail base of SL9-CpG lipovaccines (5 μg/mL SL9-FITC, 30 μg/mL CpG) or equivalent dose of controls, including physiological saline, free SL9, SL9 and cholesterol-CpG on day 10, 16, 22, and 28 after inoculation. The doses of SL9 and CpG per mouse were 500 ng and 3 μg, respectively. The tumors were measured with the caliper every two days. Tumor volumes were calculated as follows: length×width2/2. The mice were sacrificed when the tumor volume reached 1000 mm^3^ in accordance with ethics requirements. Therefore, the endpoint of the experiment was set on day 34. In addition, the mice were sacrificed on day 34 and the tumors were isolated and weighed.

### 4.9. Statistical Analysis

The data are represented as the mean ± standard deviation. Analysis of variance (ANOVA) was used to determine the significance among groups, after which, post hoc tests with the Bonferroni correction were used for multiple comparisons between individual groups. A value of *p* < 0.05 was considered to be significant.

## Abbreviations

DLS	Dynamic Laser Scattering
TEM	Transmission Electron Microscopy
FCM	Flow Cytometry
ANOVA	Analysis of Variance
PBS	Phosphate Buffered Saline
MWCO	Molar Weight Cut-Off
DMEM	Dulbecco’s Modified Eagle Medium
EPC	Egg Phosphatidylcholine
HBS	HEPES Buffer Solution
ELISA	Enzyme-Linked Immunosorbent Assay
SL9	SVYDFFVWL
CTLs	Cytotoxic T Lymphocytes
DCs	Dendritic Cells
TLR9	Toll-Like Receptor 9
BCA	Bicinchoninic Acid
FITC	Fluorescein Isothiocyanate
PE	Phycoerythrin
APC	Allophycocyanin
ODN	Oligodeoxynucleotide
CpG	Cytosine Triphosphate Deoxynucleotide-phosphodiester-Guanine Triphosphate Deoxynucleotide
EPC	Egg Phosphatidylcholine
MHC I	Major Histocompatibility Complex I
